# Supplementary material for: A Novel BoHV-1-Vectored Subunit RVFV Vaccine Induces a Robust Humoral and Cell-Mediated Immune Response Against Rift Valley Fever in Sheep
Source: Viruses. 2025 Feb 23;17(3):304. doi: 10.3390/v17030304 (PMC11945351; doi:10.3390/v17030304)
Supplement: Supplementary file 1 [file viruses-17-00304-s001.zip › Supplementary figure F1 RVFV GnPep2A-Gc Chimeras for synthesis Ovine with description nucleotide.pdf]

5’KpnI
GGTACCtagttattaatagtaatcaattacggggtcattagttcatagcccatatatggagttccgcgttacataac
CMV immediate-early promoter
Ttacggtaaatggcccgcctggctgaccgcccaacgacccccgcccattgacgtcaataatgacgtatgttcccata
gtaacgccaatagggactttccattgacgtcaatgggtggagtatttacggtaaactgcccacttggcagtacatca
agtgtatcatatgccaagtacgccccctattgacgtcaatgacggtaaatggcccgcctggcattatgcccagtaca
tgaccttatgggactttcctacttggcagtacatctacgtattagtcatcgctattaccatggtgatgcggttttgg
cagtacatcaatgggcgtggatagcggtttgactcacggggatttccaagtctccaccccattgacgtcaatgggag
tttgttttggcaccaaaatcaacgggactttccaaaatgtcgtaacaactccgccccattgacgcaaatgggcggta
ggcgtgtacggtgggaggtctatataagcagagctggtttagtgaaccgtcagatccgctagcgctaccggt**gccgc**
Kozak sequence
caccatgcagggccctaccctggctgtgctgggagctctgctggccgtggccgtgagcctcatggaggaccctcacc
BoHV-1 gD signal peptide
tgaggaaccgtccaggcaagggccacaactacatcgacggcatgacccaggaagacgctacctgcaagcccgtgacc
RVFV Gn without the TM domain
tacgcaggcgcctgcagtagtttcgacgtgctgctggagaagggcaagtttcctctgtttcagagctacgcacatca
tcgtaccctcctggaagccgtgcatgacaccatcatcgccaaggccgatcctcccagctgcgatctgctgtctgccc
acggaaacccttgcatgaaggaaaagctggtgatgaagacccattgcccaaacgactaccagagcgctcattacctc
aacaacgacggcaagatggctagcgtgaagtgccctcccaagtacgagctgaccgaggactgcaacttttgcagaca
gatgaccggagcaagcctgaagaagggatcctacccactgcaggacctcttctgccagtccagtgaggatgacggca
gcaagctgaagaccaagatgaagggtgtgtgcgaggtgggggtgcaggctctgaagaagtgcgacggccagctgagc
accgcccatgaggtggtgcctttcgctgtgttcaaaaacagcaaaaaggtgtacctcgacaagctggacctgaagac
cgaggagaacctgctgcctgacagctttgtgtgcttcgagcataagggccagtataaggggaccatggactctggac
aaaccaagcgggagctgaagagcttcgacatcagtcagtgcccaaagatcggcggacacggtagcaagaagtgcacc
ggcgacgctgccttctgcagcgcttacgagtgcaccgcccagtacgccaacgcctactgctcccacgccaacggcag
cggcatcgtgcagatccaggtgagcggcgtgtggaagaagcccctgtgcgtgggatacgagcgcgtggtggtgaagc
gcgagctcagtgctaagcctatccagcgggtggagccctgcaccacctgcatcaccaagtgcgagcctcacggcctg
gtggtgcgaagtaccggcttcaagatcagtagcgccgtggcctgcgccagtggagtgtgcgtgacaggcagtcagag
tcccagcaccgagatcacactgaagtaccctggcatctcccagtccagcggcggtgacatcggcgtgcatatggccc
atgacgatcagagtgtgagttctaagatcgtggctcattgccccccccaggacccctgcctggtgcatgactgcatc
gtgtgcgctcatggtctgatcaactaccagtgccacacccgcgtgctgaagtgcctgaagatcgcccctaggaaggt
gctgaaccctctcatgtggatcaccgcttttatcaggtggatctacaagaaaatggtggccagggtggctgacaaca
tcaaccaggtgaaccgcgagatcggatggatggaaggaggtcagctggtgctggggaacccagccccatacccgt
catgcccctatccctaga*atggctcctacccgccagcccagccccgtgactcgaccttggcagcatgtggacgctat*
Ovine GMCSF
caaggaggccctgagcctgctgaacgactccaccgacaccgcagccgtgatggacgaaaccgtggaggtggtgagcg
aaatgttcgacagtcaggagccaacctgcctgcagacccgcctggaactgtacaagcagggcctccggggaagtctc
acctccctgaccggcagtctgaccatgatggccagccattacaagaagcattgtcccccacccaggagaccagctg
cgaaactcagatcatcaccttcaagagtttcaaggagaacctgaaggacttcctgttcatcatccctttcgactgct
*gggagcccgtgcag*aaggactacaaagacgatgacgac**aagggcagcggggctaccaacttcagcctcctcaagcag**
FLAG tag **GSG + Peptide 2A**
gccggagacgtggaggaaaaccccggtcccatgtgcagcgagctgatccaggccagcagtcgaatcaccacctgcag
RVFV Gc with TM domain
caccgagggagtgaacaccaagtgccgcctgagcggcaccgcactgatccgagccggaagtgtgggcgccgaggctt
gcctgatgctgaagggcgtgaaggaggaccagaccaagttcctcaaaatcaaaaccgtgagctctgagctgagttgc
agggagggtcagagttattggaccggatccttcagtcccaaatgcctgagtagcagacggtgtcatctggtgggtga
atgccacgtgaaccgctgcctgtcctggagggacaacgagaccagtgccgagttctcttttgtgggtgagtccacca
ccatgagagagaacaagtgcttcgagcagtgcggtggatggggctgcggttgctttaacgtgaatcccagttgcctc
ttcgtgcatacctacctgcagtctgtgagaaaggaggcactgcgcgtgttcaactgcatcgactgggtgcacaaact
gaccctggagatcaccgacttcgacggaagtgtgagtaccatcgacctcggcgcctctagcagcaggtttaccaact
ggggctccgtgagtctctccctggacgccgaggggatctccggcagcaacagttttagcttcatcgagagccctggc
aagggatacgctatcgtggacgagcccttcagtgagatccctcggcagggcttcctgggcgagatccggtgcaacag
cgagagtagtgtcctgagcgctcacgagagctgcctcagagcccctaacctcatcagttacaagcctatgatcgacc
agctggagtgcaccaccaacctcattgaccctttgtggtctttgagcgcggcagtctgccccagaccaggaacgac
aagaccttcgccgccagcaagggcaaccgcggcgtgcaggccttttccaagggtagtgtgcaggccgacctcaccct
gatgttcgacaacttcgaggtggactttgtgggcgcagcagtgtcctgcgacgccgccttcctgaacctgaccggct
gttacagctgcaacgccggcgctagagtgtgcctcagcatcaccagcaccggaaccggcagcctgtccgctcataac

aaggacggcagtctgcacattgtgctgcccagcgagaacggcacaaaggaccagtgccagatcctgcacttcaccgt
gcctgaggtggaagaggaattcatgtatagctgcgacggcgacgaaaggcccctcctcgtgaagggaaccctgatcg
ccatcgatccattcgacgaccgtagagaagccggaggcgagagtaccgtggtcaaccccaagagcggaagttggaac
ttcttcgactggttttccgggctgatgagctggtttgggggcccccctgaagaccatcctgctcatctgcctgtatgt
ggccctgtccatcggactgtttttcctgctcatctacctcggtaggaccggtctgagcaaaatgtggctggccgcca
ccaagaaagcctctggtaagcctatccctaaccctctcctcggtctcgattctacgcgtaccggt**TAA***CTGATCATA*

V5 epitope tag **stop codon**

ATCAGCCATACCACATTTGTAGAGGTTTTACTTGCTTTAAAAAACCTCCCACACCTCCCCCTGAACCTGAAACATAA

SV polyA tail sequence

AATGAATGCAATTGTTGTTGTTAACTTGTTTATTGCAGCTTATAATGGTTACAAATAAAGCAATAGCATCACAAATT
*TCACAAATAAAGCATTTTTTTCACTGCATTCTAGTTGTGGTTTGTCCAAACTCATCAATGTATCTTA***AAGCTT**

HindIII-3'

Supplementrary Figure S1: Sheep-codon-optimized RVFV Gn-FLAG-P2A-Gc-V5 chimera nucleotide sequence. Rift Valley fever virus (RVFV) Gn and Gc sequence with the KpnI and HindIII sites were designed to include 5'-3' the following: Kpn1 site, cytomegalovirus (CMV) immediate-early promoter, Kozak sequence, bovine herpesvirus type 1 (BoHV-1) glycoprotein D (gD) signal peptide, RVFV Gn without the transmembrane (TM) domain, Gn cytoplasmic tail, Ovine GMCSF, FLAG tag, GSG sequence to improve cleavage efficiency, Peptide 2 A sequence, RVFV Gc region with transmembrane domain, V5 epitope, stop codon, SV polyA tail sequence and HindIII site.
