# Supplementary material for: A Novel BoHV-1-Vectored Subunit RVFV Vaccine Induces a Robust Humoral and Cell-Mediated Immune Response Against Rift Valley Fever in Sheep
Source: Viruses. 2025 Feb 23;17(3):304. doi: 10.3390/v17030304 (PMC11945351; doi:10.3390/v17030304)
Supplement: Supplementary file 1 [file viruses-17-00304-s001.zip › viruses-3467004-supplementary.pptx]

## Slide 1
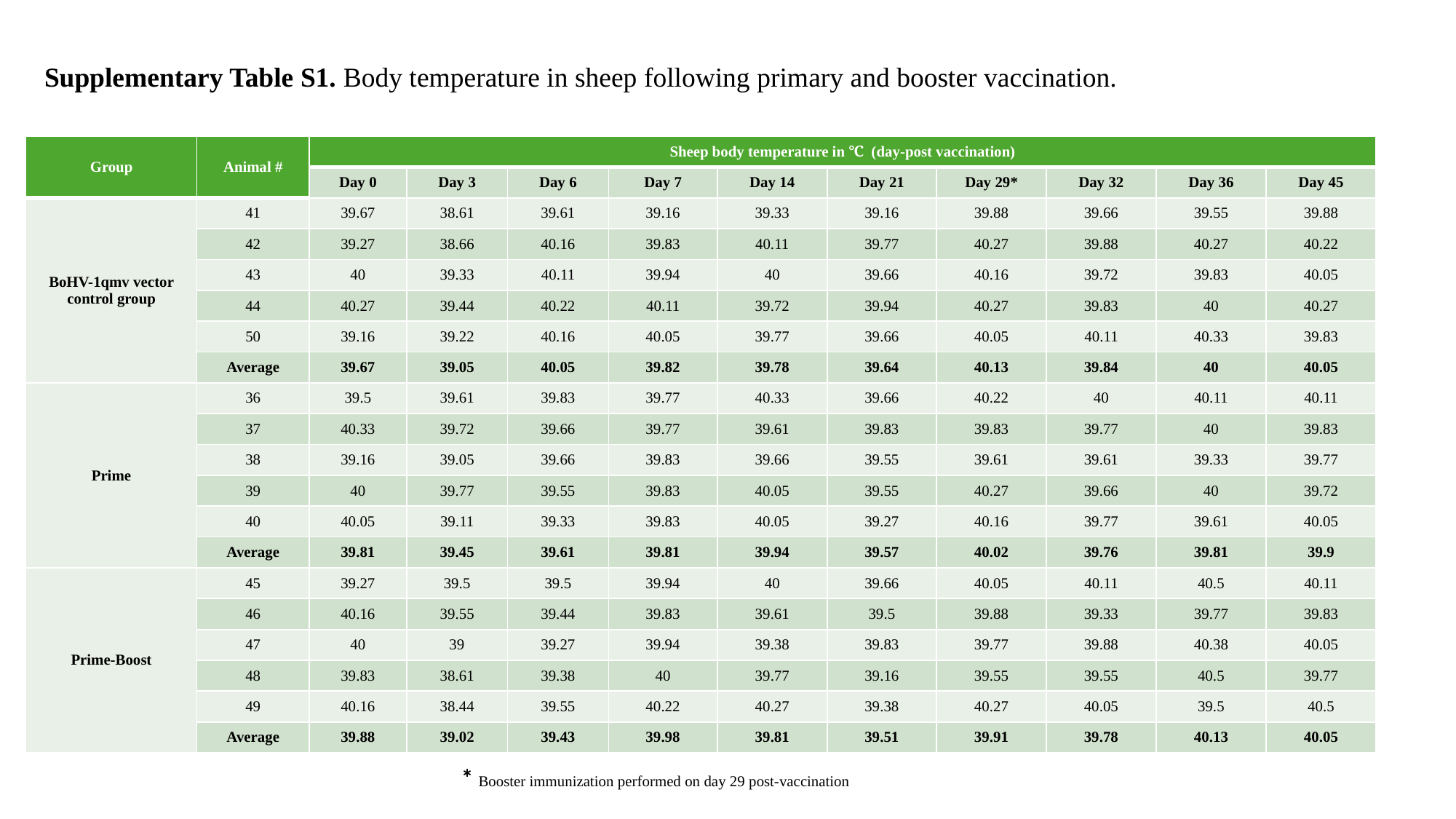

Supplementary Table S1. Body temperature in sheep following primary and booster vaccination.
| Group | Animal # | Sheep body temperature in ℃ (day-post vaccination) | | | | | | | | | |
| --- | --- | --- | --- | --- | --- | --- | --- | --- | --- | --- | --- |
| | | Day 0 | Day 3 | Day 6 | Day 7 | Day 14 | Day 21 | Day 29\* | Day 32 | Day 36 | Day 45 |
| BoHV-1qmv vector control group | 41 | 39.67 | 38.61 | 39.61 | 39.16 | 39.33 | 39.16 | 39.88 | 39.66 | 39.55 | 39.88 |
| | 42 | 39.27 | 38.66 | 40.16 | 39.83 | 40.11 | 39.77 | 40.27 | 39.88 | 40.27 | 40.22 |
| | 43 | 40 | 39.33 | 40.11 | 39.94 | 40 | 39.66 | 40.16 | 39.72 | 39.83 | 40.05 |
| | 44 | 40.27 | 39.44 | 40.22 | 40.11 | 39.72 | 39.94 | 40.27 | 39.83 | 40 | 40.27 |
| | 50 | 39.16 | 39.22 | 40.16 | 40.05 | 39.77 | 39.66 | 40.05 | 40.11 | 40.33 | 39.83 |
| | Average | 39.67 | 39.05 | 40.05 | 39.82 | 39.78 | 39.64 | 40.13 | 39.84 | 40 | 40.05 |
| Prime | 36 | 39.5 | 39.61 | 39.83 | 39.77 | 40.33 | 39.66 | 40.22 | 40 | 40.11 | 40.11 |
| | 37 | 40.33 | 39.72 | 39.66 | 39.77 | 39.61 | 39.83 | 39.83 | 39.77 | 40 | 39.83 |
| | 38 | 39.16 | 39.05 | 39.66 | 39.83 | 39.66 | 39.55 | 39.61 | 39.61 | 39.33 | 39.77 |
| | 39 | 40 | 39.77 | 39.55 | 39.83 | 40.05 | 39.55 | 40.27 | 39.66 | 40 | 39.72 |
| | 40 | 40.05 | 39.11 | 39.33 | 39.83 | 40.05 | 39.27 | 40.16 | 39.77 | 39.61 | 40.05 |
| | Average | 39.81 | 39.45 | 39.61 | 39.81 | 39.94 | 39.57 | 40.02 | 39.76 | 39.81 | 39.9 |
| Prime-Boost | 45 | 39.27 | 39.5 | 39.5 | 39.94 | 40 | 39.66 | 40.05 | 40.11 | 40.5 | 40.11 |
| | 46 | 40.16 | 39.55 | 39.44 | 39.83 | 39.61 | 39.5 | 39.88 | 39.33 | 39.77 | 39.83 |
| | 47 | 40 | 39 | 39.27 | 39.94 | 39.38 | 39.83 | 39.77 | 39.88 | 40.38 | 40.05 |
| | 48 | 39.83 | 38.61 | 39.38 | 40 | 39.77 | 39.16 | 39.55 | 39.55 | 40.5 | 39.77 |
| | 49 | 40.16 | 38.44 | 39.55 | 40.22 | 40.27 | 39.38 | 40.27 | 40.05 | 39.5 | 40.5 |
| | Average | 39.88 | 39.02 | 39.43 | 39.98 | 39.81 | 39.51 | 39.91 | 39.78 | 40.13 | 40.05 |
* Booster immunization performed on day 29 post-vaccination

## Slide 2
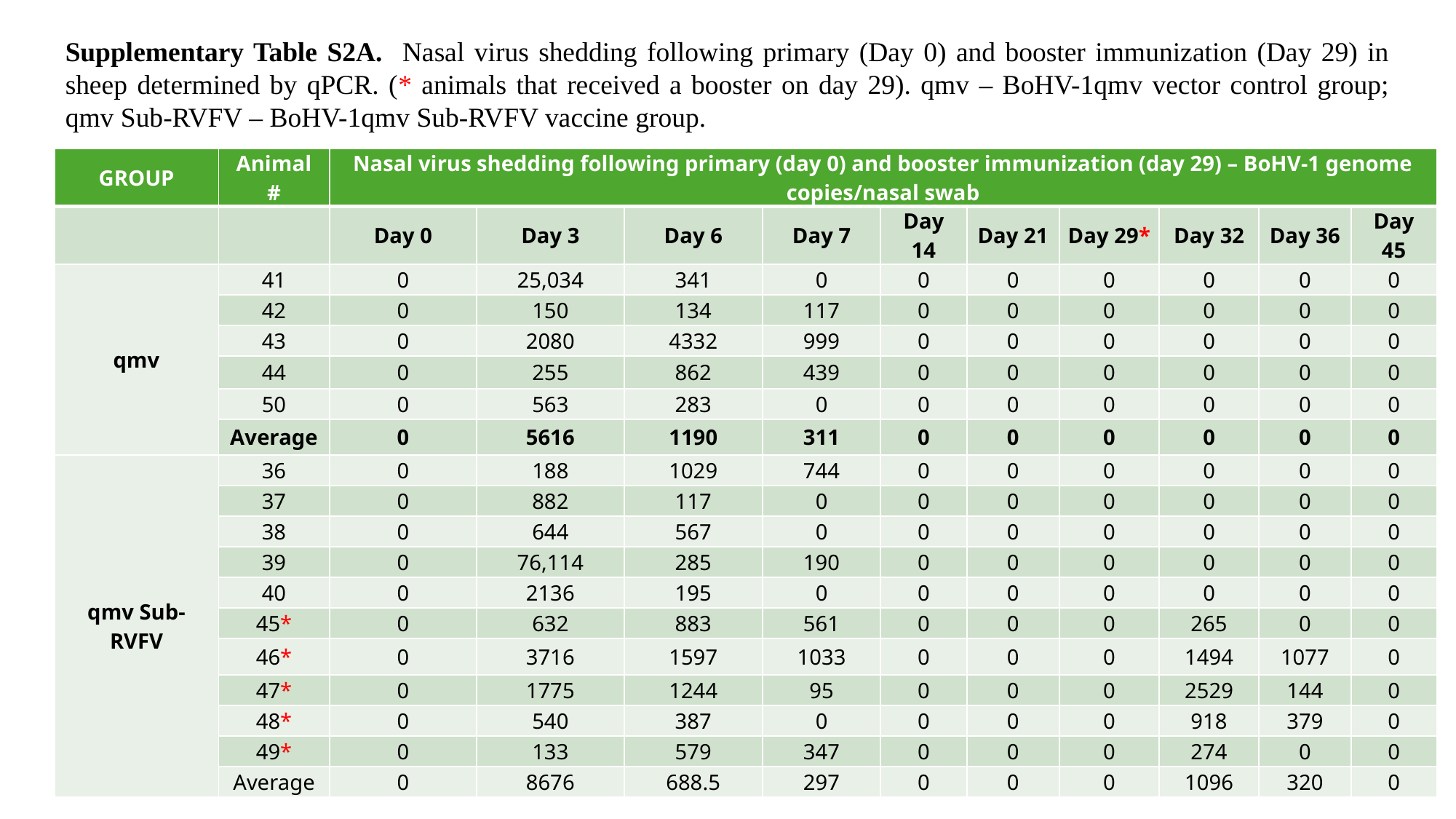

Supplementary Table S2A. Nasal virus shedding following primary (Day 0) and booster immunization (Day 29) in sheep determined by qPCR. (* animals that received a booster on day 29). qmv – BoHV-1qmv vector control group; qmv Sub-RVFV – BoHV-1qmv Sub-RVFV vaccine group.
| Group | Animal # | Nasal virus shedding following primary (day 0) and booster immunization (day 29) – BoHV-1 genome copies/nasal swab | | | | | | | | | |
| --- | --- | --- | --- | --- | --- | --- | --- | --- | --- | --- | --- |
| | | Day 0 | Day 3 | Day 6 | Day 7 | Day 14 | Day 21 | Day 29\* | Day 32 | Day 36 | Day 45 |
| qmv | 41 | 0 | 25,034 | 341 | 0 | 0 | 0 | 0 | 0 | 0 | 0 |
| | 42 | 0 | 150 | 134 | 117 | 0 | 0 | 0 | 0 | 0 | 0 |
| | 43 | 0 | 2080 | 4332 | 999 | 0 | 0 | 0 | 0 | 0 | 0 |
| | 44 | 0 | 255 | 862 | 439 | 0 | 0 | 0 | 0 | 0 | 0 |
| | 50 | 0 | 563 | 283 | 0 | 0 | 0 | 0 | 0 | 0 | 0 |
| | Average | 0 | 5616 | 1190 | 311 | 0 | 0 | 0 | 0 | 0 | 0 |
| qmv Sub-RVFV | 36 | 0 | 188 | 1029 | 744 | 0 | 0 | 0 | 0 | 0 | 0 |
| | 37 | 0 | 882 | 117 | 0 | 0 | 0 | 0 | 0 | 0 | 0 |
| | 38 | 0 | 644 | 567 | 0 | 0 | 0 | 0 | 0 | 0 | 0 |
| | 39 | 0 | 76,114 | 285 | 190 | 0 | 0 | 0 | 0 | 0 | 0 |
| | 40 | 0 | 2136 | 195 | 0 | 0 | 0 | 0 | 0 | 0 | 0 |
| | 45\* | 0 | 632 | 883 | 561 | 0 | 0 | 0 | 265 | 0 | 0 |
| | 46\* | 0 | 3716 | 1597 | 1033 | 0 | 0 | 0 | 1494 | 1077 | 0 |
| | 47\* | 0 | 1775 | 1244 | 95 | 0 | 0 | 0 | 2529 | 144 | 0 |
| | 48\* | 0 | 540 | 387 | 0 | 0 | 0 | 0 | 918 | 379 | 0 |
| | 49\* | 0 | 133 | 579 | 347 | 0 | 0 | 0 | 274 | 0 | 0 |
| | Average | 0 | 8676 | 688.5 | 297 | 0 | 0 | 0 | 1096 | 320 | 0 |

## Slide 3
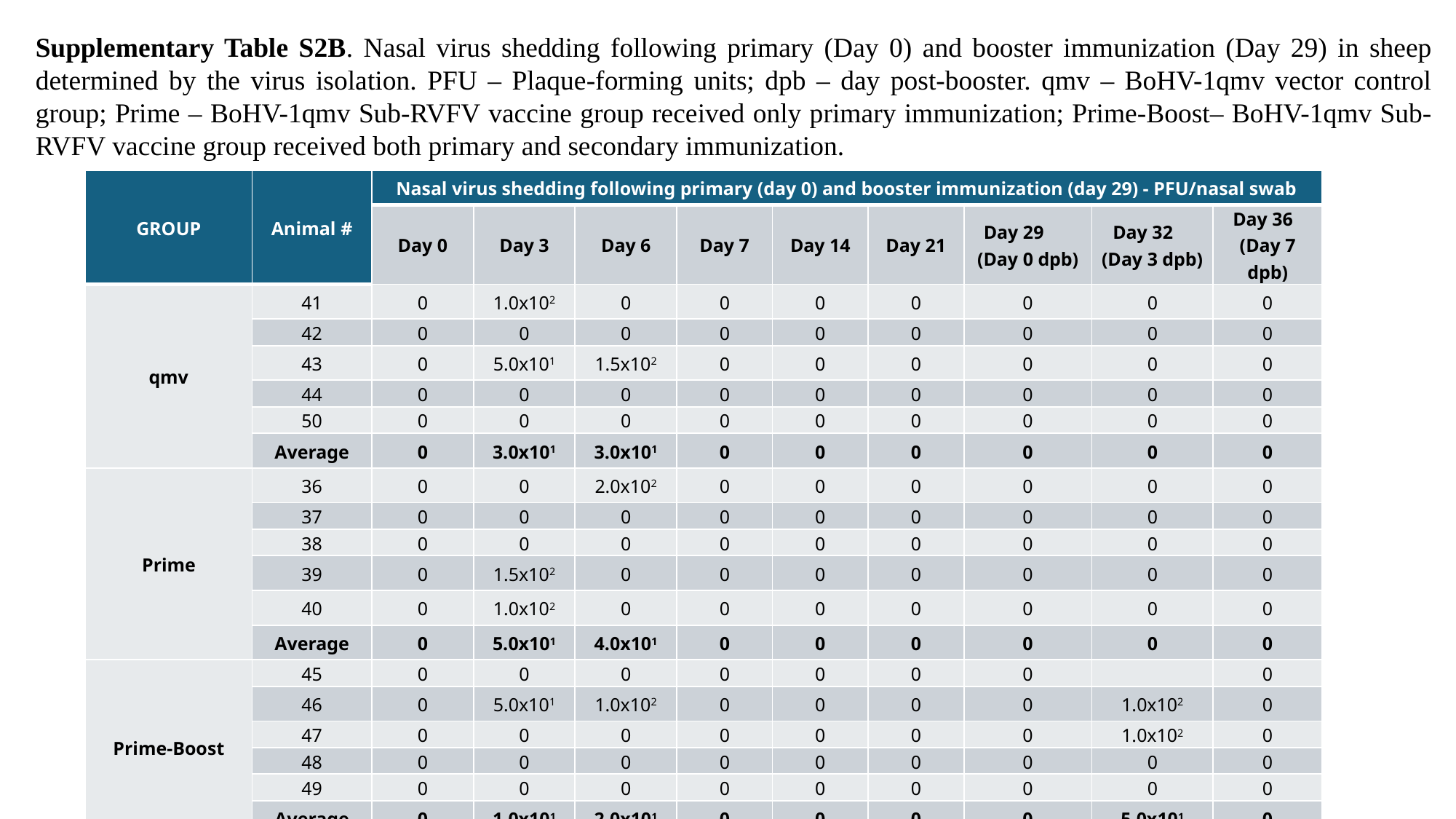

Supplementary Table S2B. Nasal virus shedding following primary (Day 0) and booster immunization (Day 29) in sheep determined by the virus isolation. PFU – Plaque-forming units; dpb – day post-booster. qmv – BoHV-1qmv vector control group; Prime – BoHV-1qmv Sub-RVFV vaccine group received only primary immunization; Prime-Boost– BoHV-1qmv Sub-RVFV vaccine group received both primary and secondary immunization.
| GROUP | Animal # | Nasal virus shedding following primary (day 0) and booster immunization (day 29) - PFU/nasal swab | | | | | | | | |
| --- | --- | --- | --- | --- | --- | --- | --- | --- | --- | --- |
| | | Day 0 | Day 3 | Day 6 | Day 7 | Day 14 | Day 21 | Day 29 (Day 0 dpb) | Day 32 (Day 3 dpb) | Day 36 (Day 7 dpb) |
| qmv | 41 | 0 | 1.0x10^2^ | 0 | 0 | 0 | 0 | 0 | 0 | 0 |
| | 42 | 0 | 0 | 0 | 0 | 0 | 0 | 0 | 0 | 0 |
| | 43 | 0 | 5.0x101 | 1.5x10^2^ | 0 | 0 | 0 | 0 | 0 | 0 |
| | 44 | 0 | 0 | 0 | 0 | 0 | 0 | 0 | 0 | 0 |
| | 50 | 0 | 0 | 0 | 0 | 0 | 0 | 0 | 0 | 0 |
| | Average | 0 | 3.0x101 | 3.0x101 | 0 | 0 | 0 | 0 | 0 | 0 |
| Prime | 36 | 0 | 0 | 2.0x10^2^ | 0 | 0 | 0 | 0 | 0 | 0 |
| | 37 | 0 | 0 | 0 | 0 | 0 | 0 | 0 | 0 | 0 |
| | 38 | 0 | 0 | 0 | 0 | 0 | 0 | 0 | 0 | 0 |
| | 39 | 0 | 1.5x10^2^ | 0 | 0 | 0 | 0 | 0 | 0 | 0 |
| | 40 | 0 | 1.0x10^2^ | 0 | 0 | 0 | 0 | 0 | 0 | 0 |
| | Average | 0 | 5.0x101 | 4.0x101 | 0 | 0 | 0 | 0 | 0 | 0 |
| Prime-Boost | 45 | 0 | 0 | 0 | 0 | 0 | 0 | 0 | | 0 |
| | 46 | 0 | 5.0x101 | 1.0x10^2^ | 0 | 0 | 0 | 0 | 1.0x10^2^ | 0 |
| | 47 | 0 | 0 | 0 | 0 | 0 | 0 | 0 | 1.0x10^2^ | 0 |
| | 48 | 0 | 0 | 0 | 0 | 0 | 0 | 0 | 0 | 0 |
| | 49 | 0 | 0 | 0 | 0 | 0 | 0 | 0 | 0 | 0 |
| | Average | 0 | 1.0x101 | 2.0x101 | 0 | 0 | 0 | 0 | 5.0x101 | 0 |

## Slide 4
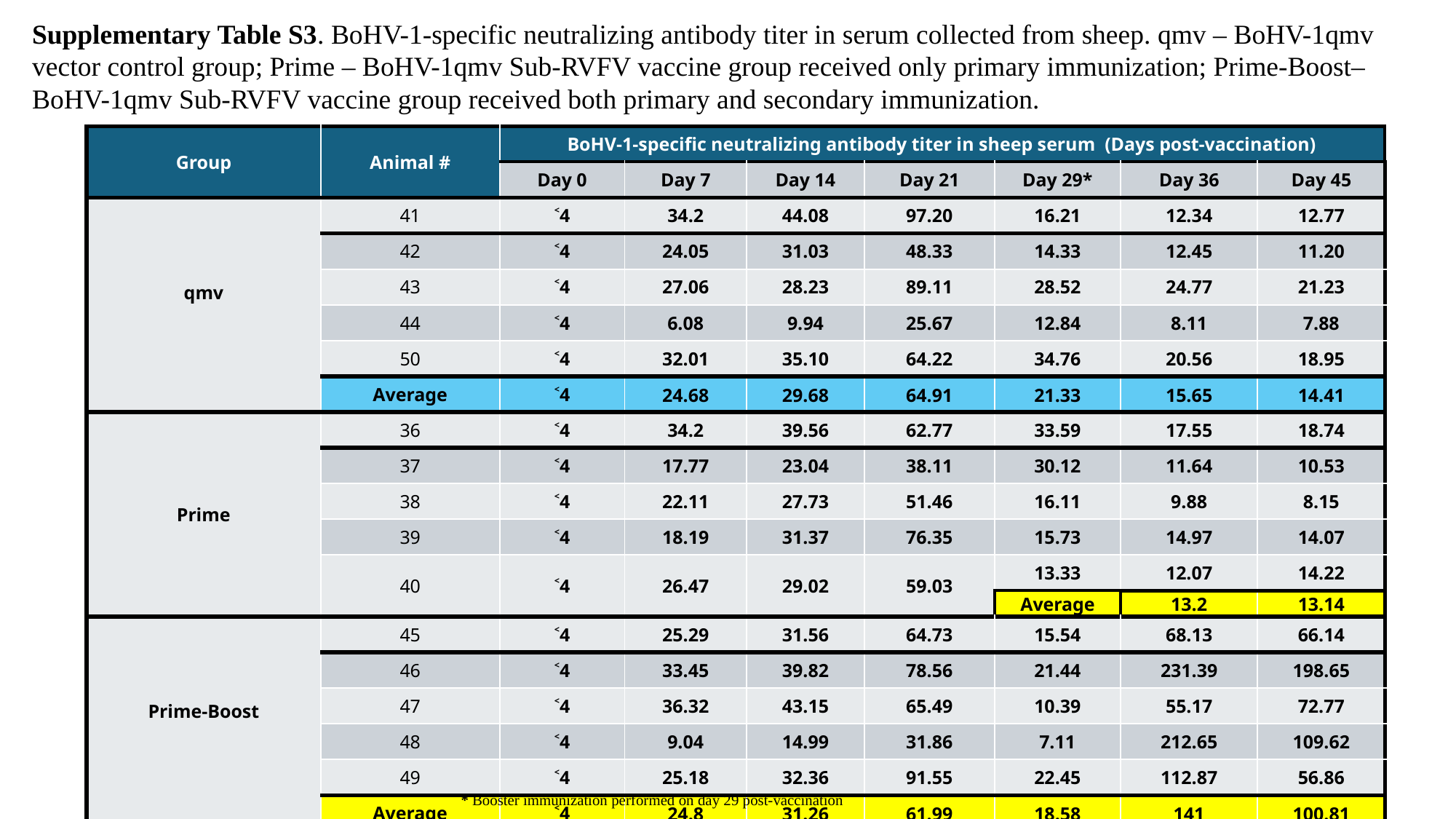

Supplementary Table S3. BoHV-1-specific neutralizing antibody titer in serum collected from sheep. qmv – BoHV-1qmv vector control group; Prime – BoHV-1qmv Sub-RVFV vaccine group received only primary immunization; Prime-Boost– BoHV-1qmv Sub-RVFV vaccine group received both primary and secondary immunization.
| Group | Animal # | BoHV-1-specific neutralizing antibody titer in sheep serum (Days post-vaccination) | | | | | | |
| --- | --- | --- | --- | --- | --- | --- | --- | --- |
| | | Day 0 | Day 7 | Day 14 | Day 21 | Day 29\* | Day 36 | Day 45 |
| qmv | 41 | ˂4 | 34.2 | 44.08 | 97.20 | 16.21 | 12.34 | 12.77 |
| | 42 | ˂4 | 24.05 | 31.03 | 48.33 | 14.33 | 12.45 | 11.20 |
| | 43 | ˂4 | 27.06 | 28.23 | 89.11 | 28.52 | 24.77 | 21.23 |
| | 44 | ˂4 | 6.08 | 9.94 | 25.67 | 12.84 | 8.11 | 7.88 |
| | 50 | ˂4 | 32.01 | 35.10 | 64.22 | 34.76 | 20.56 | 18.95 |
| | Average | ˂4 | 24.68 | 29.68 | 64.91 | 21.33 | 15.65 | 14.41 |
| Prime | 36 | ˂4 | 34.2 | 39.56 | 62.77 | 33.59 | 17.55 | 18.74 |
| | 37 | ˂4 | 17.77 | 23.04 | 38.11 | 30.12 | 11.64 | 10.53 |
| | 38 | ˂4 | 22.11 | 27.73 | 51.46 | 16.11 | 9.88 | 8.15 |
| | 39 | ˂4 | 18.19 | 31.37 | 76.35 | 15.73 | 14.97 | 14.07 |
| | 40 | ˂4 | 26.47 | 29.02 | 59.03 | 13.33 | 12.07 | 14.22 |
| | | | | | | Average | 13.2 | 13.14 |
| Prime-Boost | 45 | ˂4 | 25.29 | 31.56 | 64.73 | 15.54 | 68.13 | 66.14 |
| | 46 | ˂4 | 33.45 | 39.82 | 78.56 | 21.44 | 231.39 | 198.65 |
| | 47 | ˂4 | 36.32 | 43.15 | 65.49 | 10.39 | 55.17 | 72.77 |
| | 48 | ˂4 | 9.04 | 14.99 | 31.86 | 7.11 | 212.65 | 109.62 |
| | 49 | ˂4 | 25.18 | 32.36 | 91.55 | 22.45 | 112.87 | 56.86 |
| | Average | ˂4 | 24.8 | 31.26 | 61.99 | 18.58 | 141 | 100.81 |
* Booster immunization performed on day 29 post-vaccination

## Slide 5
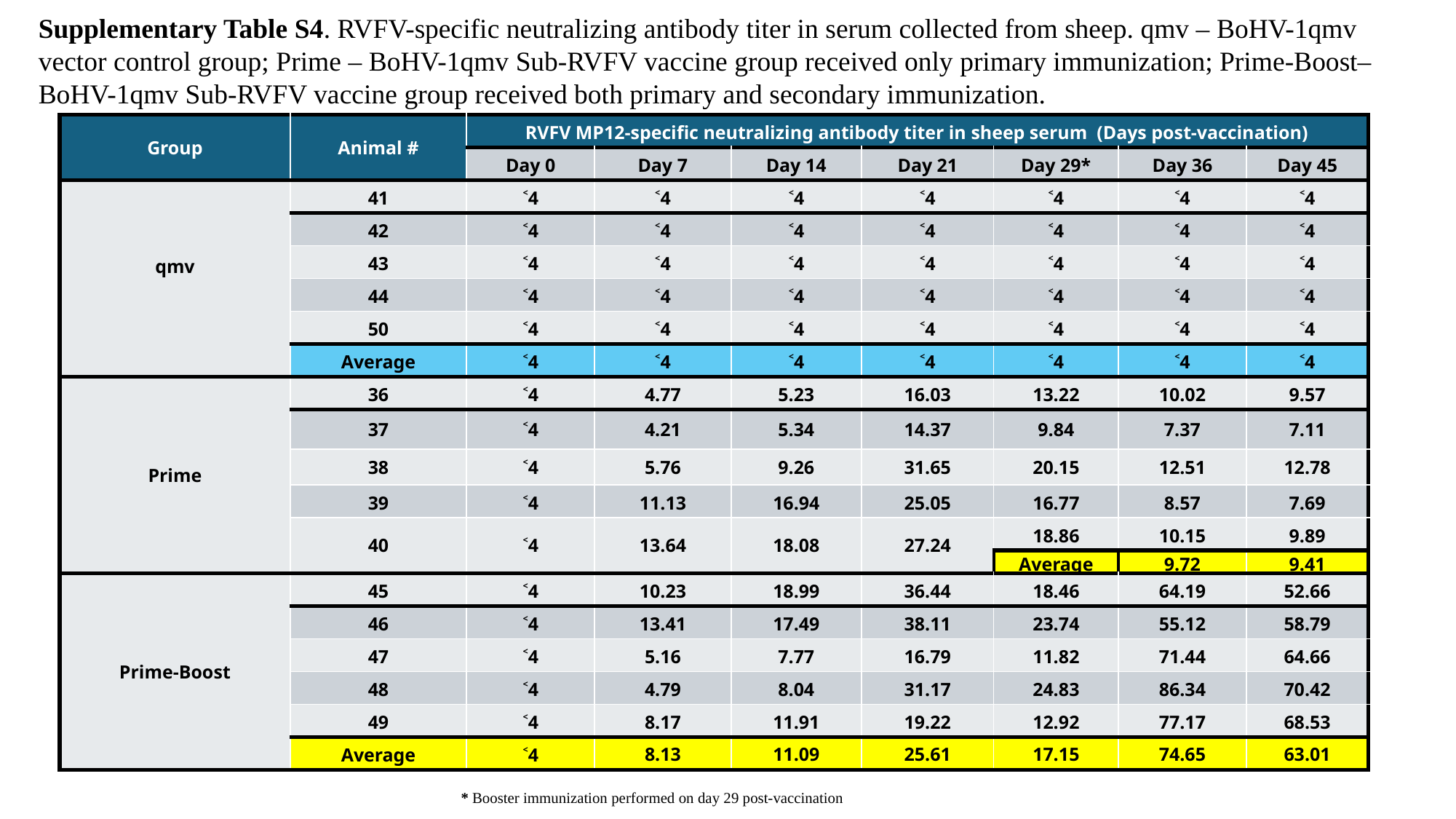

Supplementary Table S4. RVFV-specific neutralizing antibody titer in serum collected from sheep. qmv – BoHV-1qmv vector control group; Prime – BoHV-1qmv Sub-RVFV vaccine group received only primary immunization; Prime-Boost– BoHV-1qmv Sub-RVFV vaccine group received both primary and secondary immunization.
| Group | Animal # | RVFV MP12-specific neutralizing antibody titer in sheep serum (Days post-vaccination) | | | | | | |
| --- | --- | --- | --- | --- | --- | --- | --- | --- |
| | | Day 0 | Day 7 | Day 14 | Day 21 | Day 29\* | Day 36 | Day 45 |
| qmv | 41 | ˂4 | ˂4 | ˂4 | ˂4 | ˂4 | ˂4 | ˂4 |
| | 42 | ˂4 | ˂4 | ˂4 | ˂4 | ˂4 | ˂4 | ˂4 |
| | 43 | ˂4 | ˂4 | ˂4 | ˂4 | ˂4 | ˂4 | ˂4 |
| | 44 | ˂4 | ˂4 | ˂4 | ˂4 | ˂4 | ˂4 | ˂4 |
| | 50 | ˂4 | ˂4 | ˂4 | ˂4 | ˂4 | ˂4 | ˂4 |
| | Average | ˂4 | ˂4 | ˂4 | ˂4 | ˂4 | ˂4 | ˂4 |
| Prime | 36 | ˂4 | 4.77 | 5.23 | 16.03 | 13.22 | 10.02 | 9.57 |
| | 37 | ˂4 | 4.21 | 5.34 | 14.37 | 9.84 | 7.37 | 7.11 |
| | 38 | ˂4 | 5.76 | 9.26 | 31.65 | 20.15 | 12.51 | 12.78 |
| | 39 | ˂4 | 11.13 | 16.94 | 25.05 | 16.77 | 8.57 | 7.69 |
| | 40 | ˂4 | 13.64 | 18.08 | 27.24 | 18.86 | 10.15 | 9.89 |
| | | | | | | Average | 9.72 | 9.41 |
| Prime-Boost | 45 | ˂4 | 10.23 | 18.99 | 36.44 | 18.46 | 64.19 | 52.66 |
| | 46 | ˂4 | 13.41 | 17.49 | 38.11 | 23.74 | 55.12 | 58.79 |
| | 47 | ˂4 | 5.16 | 7.77 | 16.79 | 11.82 | 71.44 | 64.66 |
| | 48 | ˂4 | 4.79 | 8.04 | 31.17 | 24.83 | 86.34 | 70.42 |
| | 49 | ˂4 | 8.17 | 11.91 | 19.22 | 12.92 | 77.17 | 68.53 |
| | Average | ˂4 | 8.13 | 11.09 | 25.61 | 17.15 | 74.65 | 63.01 |
* Booster immunization performed on day 29 post-vaccination

## Slide 6
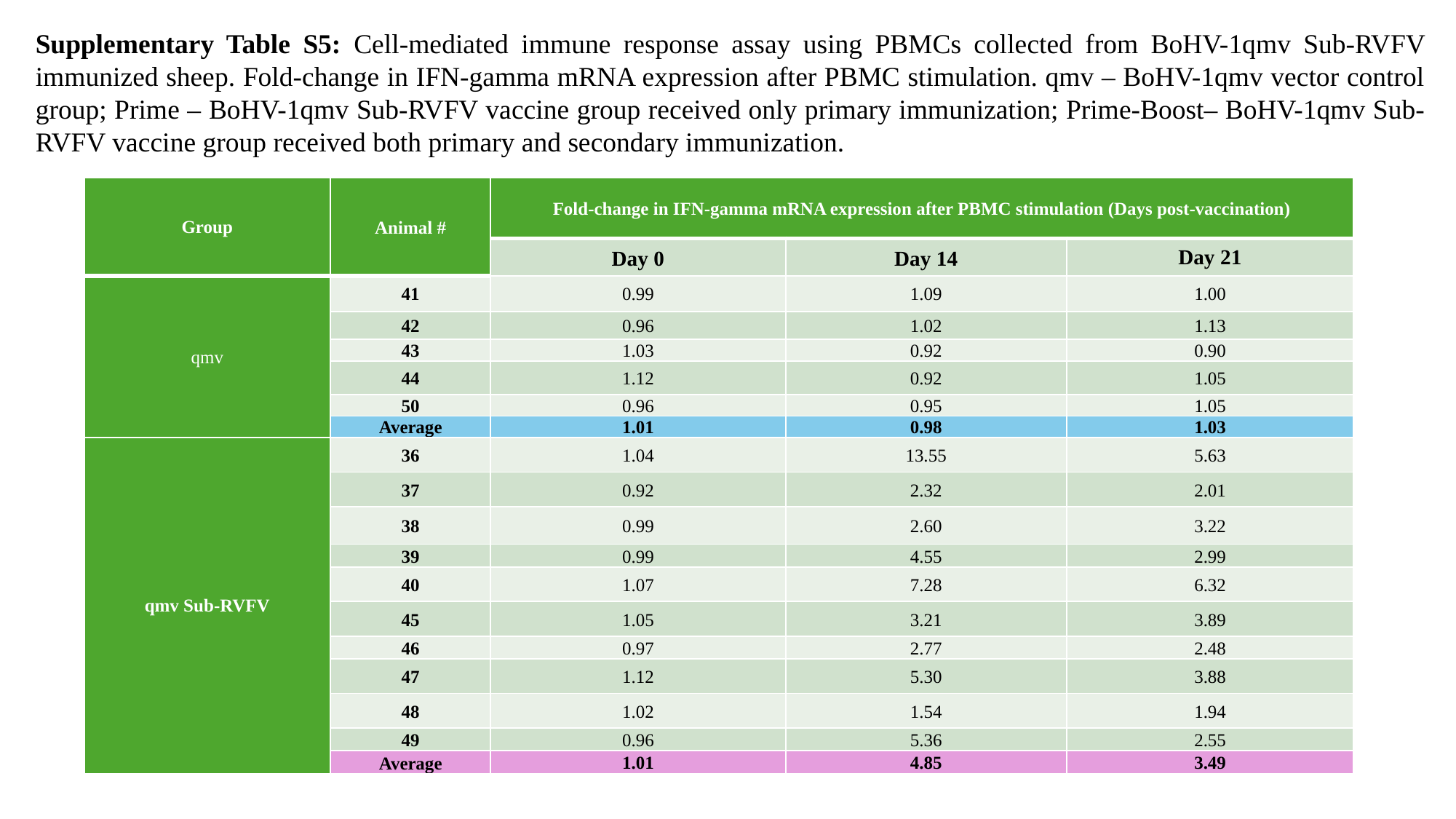

Supplementary Table S5: Cell-mediated immune response assay using PBMCs collected from BoHV-1qmv Sub-RVFV immunized sheep. Fold-change in IFN-gamma mRNA expression after PBMC stimulation. qmv – BoHV-1qmv vector control group; Prime – BoHV-1qmv Sub-RVFV vaccine group received only primary immunization; Prime-Boost– BoHV-1qmv Sub-RVFV vaccine group received both primary and secondary immunization.
| Group | Animal # | Fold-change in IFN-gamma mRNA expression after PBMC stimulation (Days post-vaccination) | | |
| --- | --- | --- | --- | --- |
| | | Day 0 | Day 14 | Day 21 |
| qmv | 41 | 0.99 | 1.09 | 1.00 |
| | 42 | 0.96 | 1.02 | 1.13 |
| | 43 | 1.03 | 0.92 | 0.90 |
| | 44 | 1.12 | 0.92 | 1.05 |
| | 50 | 0.96 | 0.95 | 1.05 |
| | Average | 1.01 | 0.98 | 1.03 |
| qmv Sub-RVFV | 36 | 1.04 | 13.55 | 5.63 |
| | 37 | 0.92 | 2.32 | 2.01 |
| | 38 | 0.99 | 2.60 | 3.22 |
| | 39 | 0.99 | 4.55 | 2.99 |
| | 40 | 1.07 | 7.28 | 6.32 |
| | 45 | 1.05 | 3.21 | 3.89 |
| | 46 | 0.97 | 2.77 | 2.48 |
| | 47 | 1.12 | 5.30 | 3.88 |
| | 48 | 1.02 | 1.54 | 1.94 |
| | 49 | 0.96 | 5.36 | 2.55 |
| | Average | 1.01 | 4.85 | 3.49 |
